# Supplementary material for: Mediastinal Parathyroid Cancer
Source: Cancers (Basel). 2022 Nov 28;14(23):5852. doi: 10.3390/cancers14235852 (PMC9739626; doi:10.3390/cancers14235852)
Supplement: Supplementary file 1 [file cancers-14-05852-s001.zip › cancers-2038037-MPC_Supplementary Datasheet S1.pdf]

Supplementary datasheet S1

436 cases of mediastinal parathyroid adenomas were identified from 221 including 41 small case series [1-41] and 179 case reports [42-221] published between 1980-2020 A hand search of articles' bibliography was also performed using the ISI Thompson Web of Knowledge Citation report to identify additional patients. Patients with secondary hyperparathyroidism were excluded . A Google Scholar search did not identify any further articles.

1. Hentati, A. and D. Gossot, *Thoracoscopic partial thymectomy for untraceable mediastinal parathyroid adenomas.* Interact Cardiovasc Thorac Surg, 2011. **13**(5): p. 542-4.
2. Binnetoglu, A., B. Demir, and J. Mamadli, *Challenges in the Management of Ectopic Parathyroid Pathologies: A Case Series of Five Patients.* Turk Arch Otorhinolaryngol, 2020. **58**(2): p. 133-136.
3. Burger, A.E., A.I. Skene, and N.R. Lagattolla, *Thoracoscopic excision of mediastinal parathyroid adenomas: a report of two cases.* Ann R Coll Surg Engl, 2008. **90**(2): p. W1-5.
4. Ward, A.F., et al., *Robot-assisted complete thymectomy for mediastinal ectopic parathyroid adenomas in primary hyperparathyroidism.* J Robot Surg, 2017. **11**(2): p. 163-169.
5. Caixas, A., et al., *Utility of 99mTc-sestamibi scintigraphy as a first-line imaging procedure in the preoperative evaluation of hyperparathyroidism.* Clin Endocrinol (Oxf), 1995. **43**(5): p. 525-30.
6. Medrano, C., et al., *Thoracoscopic resection of ectopic parathyroid glands.* Ann Thorac Surg, 2000. **69**(1): p. 221-3.
7. Rubello, D., et al., *Determinant role of Tc-99m MIBI SPECT in the localization of a retrotracheal parathyroid adenoma successfully treated by radioguided surgery.* Clin Nucl Med, 2002. **27**(10): p. 711-5.
8. Abbas, F., et al., *Mediastinal parathyroid adenoma causing primary hyperparathyroidism.* J Pak Med Assoc, 2007. **57**(2): p. 93-5.
9. Meah, F.A., et al., *Primary hyperparathyroidism--a surgical review of 12 cases.* Med J Malaysia, 1991. **46**(2): p. 144-9.
10. Mackie, G.C. and S.M. Schlicht, *Accurate localization of supernumerary mediastinal parathyroid adenomas by a combination of structural and functional imaging.* Australas Radiol, 2004. **48**(3): p. 392-7.
11. Akin, H., et al., *Mediastinal ectopic parathyroid adenoma: report of two cases successfully treated by VATS approach.* Thorac Cardiovasc Surg, 2008. **56**(1): p. 60-2.
12. Isik, H. and M. Sengul Inan, *How to do depends on where it settles: Mediastinal parathyroid adenomas.* Turk Gogus Kalp Damar Cerrahisi Derg, 2019. **28**(2): p. 340-346.
13. Nagano, H., et al., *Video-assisted thoracoscopic surgery for ectopic mediastinal parathyroid tumor: subxiphoid and lateral thoracic approach.* J Thorac Dis, 2019. **11**(7): p. 2932-2938.
14. Heller, H.J., et al., *Angiographic ablation of mediastinal parathyroid adenomas: local experience and review of the literature.* Am J Med, 1994. **97**(6): p. 529-34.
15. Curley, I.R., et al., *The challenge of the middle mediastinal parathyroid.* World J Surg, 1988. **12**(6): p. 818-24.

16. Meek, J., et al., *Percutaneous Transsternal Cryoablation of Ectopic Parathyroid Adenoma in the Anterior Mediastinum.* J Endocr Soc, 2017. **1**(10): p. 1287-1292.
17. Isaacs, K.E., et al., *Video-assisted thoracoscopic surgery for ectopic mediastinal parathyroid adenoma.* BJS Open, 2019. **3**(6): p. 743-749.
18. Cupisti, K., et al., *Therapy of suspected intrathoracic parathyroid adenomas. Experiences using open transthoracic approach and video-assisted thoracoscopic surgery.* Langenbecks Arch Surg, 2002. **386**(7): p. 488-93.
19. Amer, K., et al., *Video assisted thoracoscopic excision of mediastinal ectopic parathyroid adenomas: a UK regional experience.* Ann Cardiothorac Surg, 2015. **4**(6): p. 527-34.
20. Krudy, A.G., et al., *The detection of mediastinal parathyroid glands by computed tomography, selective arteriography, and venous sampling: an analysis of 17 cases.* Radiology, 1981. **140**(3): p. 739-44.
21. Ravipati, N.B., et al., *Anterior mediastinotomy for parathyroidectomy.* Am J Surg, 2008. **195**(6): p. 799-802.
22. Harinarayan, C.V. and G.R. Prashant, *Unusual localization of parathyroid carcinoma in anterior chest wall.* Indian J Med Res, 2013. **138**: p. 150-1.
23. Levine, D.S., A.S. Belzberg, and S.M. Wiseman, *Hybrid SPECT/CT imaging for primary hyperparathyroidism: case reports and pictorial review.* Clin Nucl Med, 2009. **34**(11): p. 779-84.
24. Berna, L., et al., *Technetium-99m-methoxyisobutylisonitrile in localization of ectopic parathyroid adenoma.* J Nucl Med, 1996. **37**(4): p. 631-3.
25. Boccalatte, L.A., et al., *[Mediastinal parathyroid adenoma detected by PET/CT with 18F choline].* Medicina (B Aires), 2018. **78**(5): p. 382.
26. Triantafyllidou, M., et al., *Localisation of ectopic mediastinal parathyroid adenoma by 18F-fluorocholine PET/CT.* BMJ Case Rep, 2018. **2018**.
27. Guerrero, M.A., et al., *Minimally invasive parathyroidectomy complicated by pneumothoraces: a report of 4 cases.* J Surg Educ, 2007. **64**(2): p. 101-7; discussion 113.
28. Kitada, M., et al., *Surgical treatment for mediastinal parathyroid adenoma causing primary hyperparathyroidism.* J Cardiothorac Surg, 2016. **11**: p. 44.
29. Iihara, M., et al., *Thoracoscopic removal of mediastinal parathyroid lesions: selection of surgical approach and pitfalls of preoperative and intraoperative localization.* World J Surg, 2012. **36**(6): p. 1327-34.
30. Ali, M. and D.A. Kumpe, *Embolization of bronchial artery-supplied ectopic parathyroid adenomas located in the aortopulmonary window.* J Vasc Interv Radiol, 2014. **25**(1): p. 138-43.
31. Muthukrishnan, J., et al., *Ectopic parathyroid adenoma--the hidden culprit.* J Assoc Physicians India, 2007. **55**: p. 515-8.
32. Downey, N.J., et al., *Median sternotomy for parathyroid adenoma.* Ir J Med Sci, 1999. **168**(1): p. 13-6.
33. Chiu, N.T., H.M. Cheng, and W.J. Yao, *Tc-99m sestamibi scanning in the preoperative localization of mediastinal parathyroid adenomas.* Ann Nucl Med, 1995. **9**(3): p. 157-9.
34. Onoda, N., et al., *Focused approach to ectopic mediastinal parathyroid surgery assisted by radio-guided navigation.* Surg Today, 2014. **44**(3): p. 533-9.
35. Toktas, O., et al., *Transcervical resection of two parathyroid adenomas located on the anterior mediastinum.* Turk J Surg, 2018. **34**(3): p. 247-249.
36. Alesina, P.F., et al., *Thoracoscopic removal of mediastinal hyperfunctioning parathyroid glands: personal experience and review of the literature.* World J Surg, 2008. **32**(2): p. 224-31.
37. Liu, R.C., M.E. Hill, and J.A. Ryan, Jr., *One-gland exploration for mediastinal parathyroid adenomas: cervical and thoracoscopic approaches.* Am J Surg, 2005. **189**(5): p. 601-4; discussion 605.

38. Phitayakorn, R. and C.R. McHenry, *Incidence and location of ectopic abnormal parathyroid glands.* Am J Surg, 2006. **191**(3): p. 418-23.
39. Wells, S.A., Jr. and J.D. Cooper, *Closed mediastinal exploration in patients with persistent hyperparathyroidism.* Ann Surg, 1991. **214**(5): p. 555-61.
40. Rabiou, S., et al., *The value of intraoperative parathyroid hormone assay in the surgery of mediastinal ectopic parathyroid adenoma (A case series).* Ann Med Surg (Lond), 2019. **44**: p. 51-56.
41. Traub-Weidinger, T., et al., *11C-methionine PET/CT imaging of 99mTc-MIBI-SPECT/CT-negative patients with primary hyperparathyroidism and previous neck surgery.* J Clin Endocrinol Metab, 2014. **99**(11): p. 4199-205.
42. Bodner, J., et al., *Mediastinal parathyroidectomy with the da Vinci robot: presentation of a new technique.* J Thorac Cardiovasc Surg, 2004. **127**(6): p. 1831-2.
43. Kirk, J., I. Au-Yong, and R. Ganatra, *Multimodality imaging of a retrosternal parathyroid adenoma with multiple brown tumors.* Clin Nucl Med, 2009. **34**(9): p. 555-9.
44. Wild, J.L., T. Weigel, and H. Chen, *The need for intraoperative parathyroid hormone monitoring during radioguided parathyroidectomy by video-assisted thoracoscopy (VATS).* Clin Nucl Med, 2006. **31**(1): p. 9-12.
45. Wei, J.P., et al., *Nonadenomatous thymic unencapsulated parathyroid tissue as a cause of persistent primary hyperparathyroidism.* South Med J, 1994. **87**(12): p. 1264-8.
46. Caravalho, J., et al., *Localization of an ectopic parathyroid adenoma by double-phase technetium-99m-sestamibi scintigraphy.* J Nucl Med, 1995. **36**(10): p. 1840-2.
47. Nakajima, J., et al., *Parathyroid adenoma manifested by mediastinal hemorrhage: report of a case.* Surg Today, 2002. **32**(9): p. 809-11.
48. Karpinski, S. and A. Sardi, *Thoracoscopic resection of a mediastinal intrathymic parathyroid adenoma.* Am Surg, 2005. **71**(12): p. 1070-2.
49. Kirkwood, I.D., et al., *Autonomous thyroid adenoma, papillary thyroid carcinoma, and ectopic parathyroid adenoma in a patient with primary hyperparathyroidism and a nontoxic multinodular goiter.* Clin Nucl Med, 1997. **22**(7): p. 491-3.
50. Long, K.L., et al., *Intrapericardial parathyroid adenomadagger.* J Surg Case Rep, 2013. **2013**(8).
51. Kumar, R., R. Gupta, and A. Malhotra, *Brown tumor of the sternum: a potential source of false-positive Tl-201 and Tc-99m subtraction imaging in the mediastinum.* Clin Nucl Med, 2000. **25**(1): p. 44-7.
52. Okuyucu, K., et al., *Parathyroid adenoma upstaging the lung cancer.* Ann Nucl Med, 2015. **29**(4): p. 371-4.
53. Tselikas, L., et al., *Microsphere and coil embolisation of a mediastinal parathyroid adenoma.* Diagn Interv Imaging, 2012. **93**(5): p. 401-5.
54. Li, L., et al., *Giant anterior mediastinal parathyroid adenoma.* Clin Nucl Med, 2012. **37**(9): p. 889-91.
55. Lunca, S., et al., *A difficult case of mediastinal parathyroid adenoma: theoretical and clinical considerations.* Chirurgia (Bucur), 2004. **99**(6): p. 563-6.
56. Chen, M., et al., *Primary hyperparathyroidism caused by mediastinal ectopic parathyroid adenoma.* Hong Kong Med J, 2017. **23**(4): p. 411-3.
57. Ebrahimpur, M., M.R. Mohajeri Tehrani, and M. Pejman Sani, *Huge Ectopic Parathyroid Adenoma.* Arch Iran Med, 2018. **21**(11): p. 547-548.
58. Abu Abeeleh, M., et al., *Aortopulmonary ectopic parathyroid gland and concurrent thymolipoma.* Asian Cardiovasc Thorac Ann, 2016. **24**(8): p. 822-824.
59. Shende, M.R., et al., *Intrathoracic parathyroid adenoma.* Ann Thorac Surg, 2004. **77**(2): p. 724.
60. Migliore, M., et al., *Persistent hyperparathyroidism owing to a giant parathyroid adenoma in posterior mediastinum.* Surgery, 2013. **154**(1): p. 132-3.

61. Heuser, M., et al., *Persistent hypocalcemia with elevated parathyroid hormone levels after long-term primary hyperparathyroidism: report of a case.* Surg Today, 2000. **30**(11): p. 1008-11.
62. Taguchi, M., et al., *Thoracoscopic findings of a small posterior mediastinal parathyroid adenoma.* Asian Cardiovasc Thorac Ann, 2006. **14**(5): p. e86-7.
63. Ishikawa, M., et al., *A rare case of mediastinal functioning parathyroid adenoma removed successfully with thoracoscopy.* J Surg Case Rep, 2017. **2017**(4): p. rjx070.
64. Mohan, M., et al., *An unusual case of brown tumor of hyperparathyroidism associated with ectopic parathyroid adenoma.* Eur J Dent, 2013. **7**(4): p. 500-503.
65. Mavai, R. and V.J. Caride, *Pin-hole collimator, parallax, and the localization of mediastinal parathyroid adenoma.* Clin Nucl Med, 1996. **21**(7): p. 527-9.
66. Uludag, M., et al., *Supernumerary ectopic parathyroid glands. Persistent hyperparathyroidism due to mediastinal parathyroid adenoma localized by preoperative single photon emission computed tomography and intraoperative gamma probe application.* Hormones (Athens), 2009. **8**(2): p. 144-9.
67. Ott, M.C., R.A. Malthaner, and R. Reid, *Intraoperative radioguided thoracoscopic removal of ectopic parathyroid adenoma.* Ann Thorac Surg, 2001. **72**(5): p. 1758-60.
68. Pecheva, M., et al., *Mediastinal giant parathyroid adenoma-a minimally invasive mediastinal surgical approach for an emergency presentation.* Ann Cardiothorac Surg, 2016. **5**(1): p. 70-3.
69. Tsuboi, M., et al., *Mediastinal Parathyroidectomy Using a Cervical Approach Under a Pneumomediastinum.* Semin Thorac Cardiovasc Surg, 2018. **30**(4): p. 472-474.
70. Siddiqi, M.S., et al., *Combined transcervical and thoracoscopic mediastinal parathyroid adenoma resection.* Asian Cardiovasc Thorac Ann, 2016. **24**(6): p. 593-6.
71. Mokrysheva, N.G., et al., *A Case of Pregnancy Complicated by Primary Hyperparathyroidism Due to a Parathyroid Adenoma.* Am J Case Rep, 2019. **20**: p. 53-59.
72. Neumann, D.R., et al., *Localization of mediastinal parathyroid adenoma in recurrent postoperative hyperparathyroidism with Tc-99m sestamibi SPECT.* Clin Nucl Med, 1995. **20**(2): p. 175.
73. Daddi, N., et al., *Transcervical resection of an ectopic mediastinal parathyroid adenoma.* Ann Thorac Surg, 2012. **94**(5): p. 1740.
74. Seniaray, N., et al., *(11)C-Methionine positron emission tomography-computed tomography in localization of methoxyisobutyl isonitrile negative ectopic parathyroid adenoma.* Indian J Nucl Med, 2016. **31**(1): p. 49-51.
75. Makay, O., et al., *Robot-assisted endoscopic mediastinal parathyroidectomy.* Turk J Surg, 2018. **34**(4): p. 315-318.
76. Lind, P., et al., *Localization of mediastinal parathyroid adenoma by T1-201 scintiscan and SPECT.* Klin Wochenschr, 1990. **68**(9): p. 472-5.
77. Moreno, P., et al., *Intercostal video-assisted mediastinal surgery through an intercostal window (IVAMS): a simpler approach to perform mediastinal parathyroidectomy.* Surgery, 2007. **142**(3): p. 410-3.
78. Garcia-Talavera, P., et al., *The value of early SPECT/CT and hand-held gamma-camera in radio-guided surgery: a case of a hard-to-locate parathyroid adenoma.* Clin Nucl Med, 2014. **39**(11): p. 1009-11.
79. Ng, P., et al., *Ectopic parathyroid adenoma localised with sestamibi SPECT and image-fused computed tomography.* Med J Aust, 2003. **179**(9): p. 485-7.
80. Thule, P., et al., *Preoperative localization of parathyroid tissue with technetium-99m sestamibi 123I subtraction scanning.* J Clin Endocrinol Metab, 1994. **78**(1): p. 77-82.
81. Kiratli, P.O., et al., *Technetium-99m pertechnetate uptake in ectopic parathyroid adenoma.* Ann Nucl Med, 1999. **13**(2): p. 113-5.

82. Pitukcheewanont, P., N. Numbenjapon, and G. Costin, *Ectopic thymic parathyroid adenoma and vitamin D deficiency rickets: a 5-year-follow-up case report and review of literature.* Bone, 2008. **42**(4): p. 819-24.
83. Batchala, P.P. and P.K. Rehm, *Retropharyngeal ectopic parathyroid adenoma versus lymph node: Problem solving with CT neck angiogram.* J Postgrad Med, 2019. **65**(4): p. 237-240.
84. Profanter, C., et al., *Robot-assisted mediastinal parathyroidectomy.* Surg Endosc, 2004. **18**(5): p. 868-70.
85. Pusuwan, P., et al., *Ectopic parathyroid imaging with Tc-99m sestamibi.* Clin Nucl Med, 1996. **21**(1): p. 74.
86. Mansberg, R., Q.L. Chiam, and C. Bui, *Radio-guided surgical excision of an ectopic mediastinal parathyroid adenoma demonstrated on Tc-99m sestamibi hybrid imaging.* Intern Med J, 2009. **39**(4): p. 263-4.
87. Welling, R.D., et al., *Bilateral retropharyngeal parathyroid hyperplasia detected with 4D multidetector row CT.* AJNR Am J Neuroradiol, 2011. **32**(5): p. E80-2.
88. Sisodiya, R., et al., *Case report on giant parathyroid adenoma with review of literature.* Indian J Surg, 2013. **75**(Suppl 1): p. 21-2.
89. Knight, R., et al., *Thoracoscopic excision of mediastinal parathyroid adenomas: a report of two cases and review of the literature.* J Am Coll Surg, 1997. **185**(5): p. 481-5.
90. Razzak, R., T. McMullen, and E.L. Bedard, *Excision of middle mediastinal parathyroid adenoma by videoscopic assisted mediastinoscopy (VAM).* J Thorac Dis, 2016. **8**(9): p. 2651-2653.
91. Iyer, R.B., G.J. Whitman, and A.A. Sahin, *Parathyroid adenoma of the mediastinum.* AJR Am J Roentgenol, 1999. **173**(1): p. 94.
92. Dogan, R., et al., *The use of gamma probe for the intraoperative localization of an ectopic parathyroid adenoma.* Tuberk Toraks, 2009. **57**(2): p. 208-11.
93. Strother, R.K. and M. Meunier, *Hypercalcemia in the Presence of an Ectopic Mediastinal Mass.* J Prim Care Community Health, 2020. **11**: p. 2150132720932411.
94. Yadav, R., et al., *Case of the season: ectopic parathyroid adenoma in the pericardium: a report of robotically assisted minimally invasive parathyroidectomy.* Semin Roentgenol, 2010. **45**(1): p. 53-6.
95. Akin, R.D. and A.D. Pinheiro, *Hypercalcemic Crisis Secondary to a Superior Mediastinal Parathyroid Adenoma: A Case Report.* Ear Nose Throat J, 2022. **101**(1): p. NP1-NP3.
96. Salido, S., et al., *Parathyroid adenoma in third pharyngeal pouch cyst as a rare case of primary hyperparathyroidism.* Ann R Coll Surg Engl, 2014. **96**(7): p. e8-10.
97. Usmani, S., et al., *Ectopic Intrathymic Parathyroid adenoma demonstrated on Tc-99m Sestamibi SPECT-CT.* Gulf J Oncolog, 2016. **1**(21): p. 61-3.
98. Dincer, S.I., et al., *Thoracoscopic removal of a maternal mediastinal ectopic parathyroid adenoma causing neonatal hypocalcemia: a case report.* Ann Thorac Cardiovasc Surg, 2008. **14**(5): p. 325-8.
99. Wiseman, S.M., et al., *Aortopulmonary window parathyroid adenoma.* J Am Coll Surg, 2009. **209**(3): p. 412.
100. Thareja, S., et al., *Pancreatitis and hyperparathyroidism: Still a rare association!* Med J Armed Forces India, 2019. **75**(4): p. 444-449.
101. Purz, S., et al., *Visualization of ectopic parathyroid adenomas.* N Engl J Med, 2013. **369**(21): p. 2067-9.
102. Rabiou, S., et al., *An Unusual Mass of Posterior Mediastinum: A Case of Retrotracheal Parathyroid Adenoma Presenting With Primary Hyperparathyroidism.* Clin Med Insights Circ Respir Pulm Med, 2018. **12**: p. 1179548418811840.
103. Choukry, S., et al., *Concomitant occurrence of primary hyperparathyroidism (PHPT) due to mediastinal parathyroid adenoma and sublingual thyroid gland: the role of parathyroid technetium-99m-MIBI scintigraphy.* Clin Case Rep, 2017. **5**(11): p. 1882-1886.

104. Ipponsugi, S., et al., *Mediastinal parathyroid adenoma detected by 99mTc-methoxyisobutylisonitrile: report of a case.* Surg Today, 1997. **27**(1): p. 80-3.
105. Fatimi, S.H., et al., *Management of mediastinal parathyroid adenoma via minimally invasive thoracoscopic surgery: Case report.* Int J Surg Case Rep, 2017. **40**: p. 120-123.
106. Iyer, S., et al., *Retro-tracheal parathyroid adenoma: A rare location of a common pathology.* Radiol Case Rep, 2020. **15**(6): p. 672-674.
107. Liman, S.T., et al., *Excision of ectopic mediastinal parathyroid adenoma via parasternal videomediastinoscopy.* Ann Thorac Cardiovasc Surg, 2014. **20**(1): p. 67-9.
108. Bearelly, S., et al., *Transoral robotic-assisted surgical excision of a retropharyngeal parathyroid adenoma: a case report.* Head Neck, 2015. **37**(11): p. E150-2.
109. Talukder, S., et al., *Giant mediastinal parathyroid adenoma presenting as bilateral brown tumour of mandible: a rare presentation of primary hyperparathyroidism.* BMJ Case Rep, 2017. **2017**.
110. Mitsuboshi, S., et al., *Thoracoscopic surgical case of an ectopic mediastinal parathyroid adenoma detected by chance: a case report.* BMC Surg, 2019. **19**(1): p. 171.
111. Buderi, S.I., et al., *Endobronchial ultrasound-guided biopsy to diagnose large posterior mediastinal parathyroid adenoma prior to video-assisted thoracoscopic resection.* BMJ Case Rep, 2014. **2014**.
112. Jasim, S. and K. Kennel, *Persistent hyperparathyroidism due to ectopic parathyroid gland.* Endocrine, 2017. **55**(1): p. 322-323.
113. Smirniotopoulos, J., et al., *CT-Guided Cryoablation of a Substernal Mediastinal Ectopic Parathyroid Adenoma.* J Vasc Interv Radiol, 2017. **28**(4): p. 614-616.
114. Gouveia, S., et al., *Persistent primary hyperparathyroidism: an uncommon location for an ectopic gland--case report and review.* Arq Bras Endocrinol Metabol, 2012. **56**(6): p. 393-403.
115. Daliakopoulos, S.I., et al., *Gamma probe-assisted excision of an ectopic parathyroid adenoma located within the thymus: case report and review of the literature.* J Cardiothorac Surg, 2014. **9**: p. 62.
116. Urata, T., et al., *Acute pancreatitis caused by an ectopic mediastinal parathyroid adenoma.* Clin J Gastroenterol, 2012. **5**(6): p. 393-7.
117. Moriyama, T., et al., *Diagnosis of a case of ectopic parathyroid adenoma on the early image of 99mTc-MIBI scintigram.* Endocr J, 2007. **54**(3): p. 437-40.
118. Obara, T., et al., *Mid-mediastinal parathyroid lesions: preoperative localization and surgical approach in two cases.* Jpn J Surg, 1990. **20**(4): p. 481-6.
119. Nakada, T., et al., *A case of a retroesophageal parathyroid adenoma with an aberrant right subclavian artery: a potential surgical pitfall.* Ann Thorac Cardiovasc Surg, 2014. **20 Suppl**: p. 786-9.
120. Shimada, T., et al., *Magnetic resonance imaging as an effective tool for successful localization of superior mediastinal parathyroid adenoma.* Jpn J Med, 1990. **29**(6): p. 647-51.
121. Yoshida, T., et al., *Thoracoscopically managed parathyroid adenoma in the upper anterior mediastinum.* Surg Laparosc Endosc Percutan Tech, 2001. **11**(6): p. 385-8.
122. Ogawa, T., et al., *Excision of postesophageal parathyroid adenoma in posterior mediastinum with intraoperative 99mTechnetium sestamibi scanning.* Ann Thorac Surg, 2007. **84**(5): p. 1754-6.
123. Gonzalez, V.G., et al., *Early parathyroid MIBI SPECT imaging in the diagnosis of persistent hyperparathyroidism.* Clin Nucl Med, 2008. **33**(7): p. 475-8.
124. Vijayakumar, V. and M.E. Anderson, *Detection of ectopic parathyroid adenoma by early Tc-99m sestamibi imaging.* Ann Nucl Med, 2005. **19**(2): p. 157-9.
125. Dhillon, V.K., et al., *Identifying an Ectopic Parathyroid Adenoma Using 4DCT in a Pediatric Patient with Persistent Primary Hyperparathyroidism.* Case Rep Otolaryngol, 2013. **2013**: p. 676039.

126. Ayyildiz, V., M. Ay, and H. Ogul, *Ectopic mediastinal parathyroid adenoma.* Br J Hosp Med (Lond), 2020. **81**(3): p. 1.
127. Beisa, V., et al., *Thoracoscopic approach in the treatment of ectopic thymic parathyroid adenoma.* Wideochir Inne Tech Maloinwazyjne, 2018. **13**(2): p. 270-277.
128. Yoon, V., K. Treat, and N.M. Maalouf, *Ectopic atypical parathyroid lipoadenoma: a rare cause of severe primary hyperparathyroidism.* J Bone Miner Metab, 2013. **31**(5): p. 595-600.
129. Verwimp, W., R. Bracke, and H. Degryse, *Ectopic parathyroid adenoma in the upper anterior mediastinum.* JBR-BTR, 2011. **94**(3): p. 154-5.
130. Kim, W.K., et al., *Intra-thoracic Parathyroid Adenomatosis: A Case Report.* Korean J Thorac Cardiovasc Surg, 2013. **46**(4): p. 302-4.
131. Horton, W.B., et al., *Gestational Primary Hyperparathyroidism Due to Ectopic Parathyroid Adenoma: Case Report and Literature Review.* J Endocr Soc, 2017. **1**(9): p. 1150-1155.
132. Liu, X., et al., *Primary hyperparathyroidism due to ectopic parathyroid adenoma in an adolescent: a case report and review of the literature.* Endocrine, 2019. **64**(1): p. 38-42.
133. Liu, X., et al., *Comparative imaging of ectopic mediastinal parathyroid adenoma with magnetic resonance imaging and single photon emission computed tomography/computed tomography: advantages of multimodality imaging.* Korean J Intern Med, 2020. **35**(4): p. 1024-1025.
134. Wang, X., et al., *Surgery for Ectopic Parathyroid Adenoma in Lower Part of Superior Mediastinum through a Transcervical Incision.* Chin Med J (Engl), 2017. **130**(11): p. 1376-1377.
135. Krausz, Y., et al., *Lateral neck imaging for spatial localization of parathyroid tissue.* Nucl Med Biol, 1995. **22**(3): p. 391-4.
136. Minamiya, Y., et al., *Radio-guided thoracoscopic surgery with (99m)Tc-methoxy-isobutylisonitrile for treating an ectopic mediastinal parathyroid adenoma in an adolescent girl.* Gen Thorac Cardiovasc Surg, 2009. **57**(12): p. 657-9.
137. Nishimura, Y., et al., *Cognitive decline due to ectopic primary hyperparathyroidism.* Clin Case Rep, 2018. **6**(12): p. 2513-2514.
138. Kim, Y.S., J. Kim, and S. Shin, *Thoracoscopic removal of ectopic mediastinal parathyroid adenoma.* Korean J Thorac Cardiovasc Surg, 2014. **47**(3): p. 317-9.
139. Seo, Y., et al., *A case of primary hyperparathyroidism due to an intrathymic ectopic parathyroid adenoma in a 15-year-old boy.* Ann Pediatr Endocrinol Metab, 2020. **25**(3): p. 187-191.
140. Zeng, Z., et al., *Mediastinal ectopic parathyroid adenoma.* QJM, 2019. **112**(2): p. 127-128.
141. Koc, Z.P., et al., *Minimal Invasive Radioguided Ectopic Parathyroidectomy in Upper Mediastinum.* Mol Imaging Radionucl Ther, 2019. **28**(3): p. 120-122.
142. Schwarzlmuller, T., et al., *High cardiac background activity limits 99mTc-MIBI radioguided surgery in aortopulmonary window parathyroid adenomas.* BMC Surg, 2014. **14**: p. 22.
143. Wang, F., et al., *Perioperative Clinical Features of Mediastinal Parathyroid Adenoma: A Case Series.* Thorac Cardiovasc Surg, 2022.
144. Ikeda, Y., et al., *Cervical approach by lifting the superior sternum for mediastinal parathyroid adenoma.* Surg Laparosc Endosc Percutan Tech, 2009. **19**(1): p. 78-81.
145. Kang, Y.S., et al., *Localization of abnormal parathyroid glands of the mediastinum with MR imaging.* Radiology, 1993. **189**(1): p. 137-41.
146. Hiromatsu, Y., et al., *Technetium-99m tetrofosmin parathyroid imaging in patients with primary hyperparathyroidism.* Intern Med, 2000. **39**(2): p. 101-6.
147. Haldar, A., et al., *Day-case minimally invasive excision of a giant mediastinal parathyroid adenoma.* Ann R Coll Surg Engl, 2014. **96**(5): p. e21-3.
148. El Beltagi, A.H., et al., *MRI localization of mildly symptomatic ectopic retropharyngeal parathyroid adenoma: the impact on surgical approach. A case report.* Neuroradiol J, 2011. **24**(6): p. 938-41.

149. Mohammed, A.R., R. Rea, and C. Ubhi, *An unusual cause of a mediastinal mass.* BMJ Case Rep, 2010. **2010**.
150. Moller, M.L., et al., *Clinical value of 11C-methionine positron emission tomography in persistent primary hyperparathyroidism-A case report with a mediastinal parathyroid adenoma.* Int J Surg Case Rep, 2018. **45**: p. 63-66.
151. Graff-Baker, A., et al., *Diagnosis of ectopic middle mediastinal parathyroid adenoma using endoscopic ultrasonography-guided fine-needle aspiration with real-time rapid parathyroid hormone assay.* J Am Coll Surg, 2009. **209**(3): p. e1-4.
152. Andre, N., et al., *Impact of incidental parathyroidectomy and mediastinal-recurrent cellular and lymph-node dissection on parathyroid function after total thyroidectomy.* Eur Ann Otorhinolaryngol Head Neck Dis, 2020. **137**(2): p. 107-110.
153. Hillenbrand, A., et al., *Can Met-PET/CT Predict Sporadic Multiglandular Hyperparathyroidism? Report of a Case and Review of the Literature.* Case Rep Endocrinol, 2019. **2019**: p. 1791740.
154. Vaidya, A., et al., *Ectopic Parathyroid Adenoma Presenting as a Mediastinal Mass.* J Clin Diagn Res, 2017. **11**(5): p. ED40-ED42.
155. Gurrado, A., et al., *Substernal oxyphil parathyroid adenoma producing PTHrP with hypercalcemia and normal PTH level.* World J Surg Oncol, 2008. **6**: p. 24.
156. Kane, A.C., R.R. Walvekar, and J.M. Hotaling, *Transoral robotic resection of a retropharyngeal parathyroid adenoma: a case report.* J Robot Surg, 2019. **13**(2): p. 335-338.
157. Adewole, A.D., et al., *A fluorodeoxyglucose avid mediastinal parathyroid adenoma masquerading as metastatic bladder cancer.* Interact Cardiovasc Thorac Surg, 2012. **15**(3): p. 514-5.
158. Kordahi, A.M., et al., *Undescended retropharyngeal parathyroid adenoma with adjacent thymic tissue in a 13-year-old boy with primary hyperparathyroidism.* Oxf Med Case Reports, 2019. **2019**(12): p. 519-523.
159. Saad, A.F., L.D. Pacheco, and M.M. Costantine, *Management of ectopic parathyroid adenoma in pregnancy.* Obstet Gynecol, 2014. **124**(2 Pt 2 Suppl 1): p. 478-480.
160. Patrinos, A., et al., *An anatomic aberration and a surgical challenge: Mediastinal parathyroid adenoma anterior the pericardium. A case report.* Int J Surg Case Rep, 2019. **58**: p. 153-156.
161. Kumar, A., et al., *Thoracoscopy: the preferred method for excision of mediastinal parathyroids.* Surg Laparosc Endosc Percutan Tech, 2002. **12**(4): p. 295-300.
162. Gungunes, A., et al., *Nonadenomatous nonencapsulated thymic parathyroid tissue concomitant with primary hyperparathyroidism due to ectopic parathyroid adenoma.* Arq Bras Endocrinol Metabol, 2013. **57**(9): p. 739-42.
163. Yassine, A., et al., *[An unusual mode of discovery of primary hyperparathyroidism: multiple fractures on brown tumors secondary to ectopic mediastinal parathyroid adenoma].* Pan Afr Med J, 2015. **22**: p. 290.
164. Hicham, A. and M. Abdelhamid, *Hyperparathyroidism related to an ectopic parathyroid adenoma.* Pan Afr Med J, 2014. **19**: p. 135.
165. Harris, B., et al., *Use of fusion imaging to localize an ectopic thoracic parathyroid adenoma.* Ann Thorac Surg, 2006. **82**(2): p. 719-21.
166. Miller, B.J., et al., *Transcervical excision of a giant mediastinal parathyroid adenoma.* BMJ Case Rep, 2019. **12**(2).
167. Walton, B., et al., *Microscopic thymoma and parathyroid adenoma: rare combination of two distinct pathologies.* Rare Tumors, 2014. **6**(2): p. 5197.
168. Adams, B.K., R.T. Devi, and Z.Y. Al-Haider, *Tc-99m sestamibi localization of an ectopic mediastinal parathyroid tumor in a patient with primary hyperparathyroidism.* Clin Nucl Med, 2004. **29**(6): p. 388-9.

169. Whitson, B.A., et al., *Acute airway compromise from a hemorrhagic posterior cervical-mediastinal mass: Rare presentation of a parathyroid adenoma.* J Thorac Dis, 2011. **3**(1): p. 68-70.
170. Di Cosimo, C., et al., *Mediastinal parathyroid adenoma: a case report.* Eur Rev Med Pharmacol Sci, 2012. **16**(6): p. 845-7.
171. Chen, C.E., C.L. Kao, and C.J. Wang, *Bilateral pathological femoral neck fractures secondary to ectopic parathyroid adenoma.* Arch Orthop Trauma Surg, 1998. **118**(3): p. 164-6.
172. Garingarao, C.J., E. Paz-Pacheco, and C.A. Jimeno, *Primary hyperparathyroidism from a probable ectopic parathyroid adenoma with severe skeletal disease and vitamin D deficiency.* BMJ Case Rep, 2014. **2014**.
173. Corvera, C.U., et al., *Retrocardiac parathyroid tumor: a rare mediastinal site.* Surgery, 2004. **135**(1): p. 104-7.
174. Spear, C., et al., *Resection of an Ectopic Parathyroid Adenoma via Video-Assisted Mediastinoscopy.* Semin Thorac Cardiovasc Surg, 2019. **31**(2): p. 323-325.
175. Che Kadir, S., et al., *Mediastinal parathyroid adenoma: diagnostic and management challenges.* Singapore Med J, 2011. **52**(4): p. e70-4.
176. Chen, C.C., et al., *Tc-99m uptake in a parathyroid adenoma. Potential pitfall in Tc-99m/Tl-201 subtraction imaging.* Clin Nucl Med, 1992. **17**(7): p. 539-41.
177. Zhao, C., et al., *Parathyroid adenoma causing a spontaneous cervical and mediastinal massive hematoma.* Int J Clin Exp Med, 2015. **8**(11): p. 21826-9.
178. Lu, C., et al., *Superior mediastinal typical carcinoid detected by 99mTc-MIBI SPECT/CT imaging: A case report.* Medicine (Baltimore), 2017. **96**(52): p. e9457.
179. Foroulis, C.N., et al., *Ectopic paraesophageal mediastinal parathyroid adenoma, a rare cause of acute pancreatitis.* World J Surg Oncol, 2004. **2**: p. 41.
180. Batsakis, C., et al., *Giant mediastinal parathyroid adenoma in a woman with hypercalcemia.* Clin Nucl Med, 2001. **26**(11): p. 950-1.
181. Grozavu, C. and D. Pantile, *Primary Hyperparathyroidism Through an Ectopic Parathyroid Adenoma.* Chirurgia (Bucur), 2016. **111**(2): p. 156-60.
182. Rooney, D.P., et al., *Cure of hyperparathyroidism in pregnancy by sternotomy and removal of a mediastinal parathyroid adenoma.* Postgrad Med J, 1998. **74**(870): p. 233-4.
183. Axelrod, D., et al., *Appearance of ectopic undescended inferior parathyroid adenomas on technetium Tc 99m sestamibi scintigraphy: a lesson from reoperative parathyroidectomy.* Arch Surg, 2003. **138**(11): p. 1214-8.
184. Martinez, D.A., et al., *Intraoperative identification of parathyroid gland pathology: a new approach.* J Pediatr Surg, 1995. **30**(9): p. 1306-9.
185. Ergi, D.G., et al., *Targeted Minimally Invasive Parathyroidectomy for Ectopic Aortopulmonary Adenoma Under Gamma Probe Guidance.* Innovations (Phila), 2018. **13**(6): p. 451-454.
186. Arik, D., et al., *Water-Clear Cell Adenoma of the Mediastinal Parathyroid Gland.* Turk Patoloji Derg, 2019. **35**(2): p. 157-161.
187. Simeone, D.M., K. Sandelin, and N.W. Thompson, *Undescended superior parathyroid gland: a potential cause of failed cervical exploration for hyperparathyroidism.* Surgery, 1995. **118**(6): p. 949-56.
188. Piciu, D., et al., *Primary hyperparathyroidism-jaw tumor syndrome: a confusing and forgotten diagnosis.* Clujul Med, 2016. **89**(4): p. 555-558.
189. Rubello, D., et al., *Efficacy of sequential double tracer subtraction and SPECT parathyroid imaging in the precise localization of a low mediastinal parathyroid adenoma successfully removed surgically.* Clin Nucl Med, 2004. **29**(10): p. 662-3.
190. Naik, D., et al., *Ectopic thymic parathyroid adenoma.* BMJ Case Rep, 2014. **2014**.
191. Martinez-Gavidia, E.M., et al., *Highly aggressive brown tumour of the maxilla as first manifestation of primary hyperparathyroidism.* Int J Oral Maxillofac Surg, 2000. **29**(6): p. 447-9.

192. Souza, E.R., et al., *Devastating skeletal effects of delayed diagnosis of complicated primary hyperparathyroidism because of ectopic adenoma.* J Clin Rheumatol, 2008. **14**(5): p. 281-4.
193. Yesilkaya, E., et al., *Hungry bone syndrome after parathyroidectomy caused by an ectopic parathyroid adenoma.* J Bone Miner Metab, 2009. **27**(1): p. 101-4.
194. Mancilla, E.E., M.A. Levine, and N.S. Adzick, *Outcomes of minimally invasive parathyroidectomy in pediatric patients with primary hyperparathyroidism owing to parathyroid adenoma: A single institution experience.* J Pediatr Surg, 2017. **52**(1): p. 188-191.
195. Teigen, E.L., et al., *Technetium-99m-sestamibi SPECT localization of mediastinal parathyroid adenoma.* J Nucl Med, 1996. **37**(9): p. 1535-7.
196. Van Dessel, E., et al., *Mediastinal parathyroidectomy with the da Vinci robot.* Innovations (Phila), 2011. **6**(4): p. 262-4.
197. Funk, E., et al., *Presentation of an Ectopic Intrathymic Parathyroid Adenoma as a Lateral Neck Mass.* Ear Nose Throat J, 2019. **98**(2): p. 68-69.
198. Fallone, E., et al., *Ectopic (mediastinal) parathyroid adenoma with prominent lymphocytic infiltration.* Appl Immunohistochem Mol Morphol, 2009. **17**(1): p. 82-4.
199. Ersen, E. and B. Kilic, *Thoracoscopic removal of ectopic mediastinal parathyroid adenoma causing hyperparathyroidism: a rare entity.* Wideochir Inne Tech Maloinwazyjne, 2018. **13**(4): p. 546-550.
200. Qari, F.A., *Brown tumor in a patient with ectopic mediastinal parathyroid adenoma: A case report.* Saudi Dent J, 2014. **26**(2): p. 74-7.
201. El Oueriachi, F., A. Arsalane, and H. Kabiri el, *Uncommon ectopic parathyroid adenoma.* Arch Bronconeumol, 2015. **51**(6): p. 301-2.
202. Raveglia, F., et al., *Unexpected thymoma in a challenging case of hyperparathyroidism.* Clin Case Rep, 2020. **8**(8): p. 1425-1428.
203. Sellitri, F., et al., *Intrathymic ectopic parathyroid adenoma caused primary hyperparathyroidism with vitamin D deficiency several years after bariatric surgery.* Thorac Cancer, 2015. **6**(1): p. 101-4.
204. Flickinger, F.W., et al., *MRI in hyperparathyroidism requiring reoperation.* Clin Imaging, 1991. **15**(3): p. 210-2.
205. Yuce, G. and N.C. Seyrek, *Persistent hyperparathyroidism due to mediastinal parathyroid adenoma treated with selective arterial embolization with embosphere: first case in the literature.* Osteoporos Int, 2020. **31**(11): p. 2259-2262.
206. Winzelberg, G.G., *Thallium 201/99mTc parathyroid subtraction scintigraphy of the mediastinum.* Semin Nucl Med, 1987. **17**(3): p. 278-9.
207. Cook, G.J., I. Fogelman, and J.F. Reidy, *Successful repeat transcatheter ablation of a mediastinal parathyroid adenoma 6 years after alcohol embolization.* Cardiovasc Intervent Radiol, 1997. **20**(4): p. 314-6.
208. Wang, G., et al., *A case of primary hyperparathyroidism due to ectopic parathyroid adenoma in the thymus, accompanied with vitamin D deficiency.* J Clin Endocrinol Metab, 2013. **98**(6): p. 2218-22.
209. Schmidt, H., B. Kusser, and F. Spelsberg, *Asymptomatic hypercalcemia due to an ectopic parathyroid adenoma in an 8-year-old boy.* Exp Clin Endocrinol Diabetes, 2001. **109**(3): p. 184-6.
210. Taghavi Kojidi, H., et al., *Unusual Ectopic Parathyroid Adenoma: A Case Report.* Acta Med Iran, 2016. **54**(8): p. 547-550.
211. Lazar, H.L., E. Oates, and R.M. Beazley, *Excision of a mediastinal parathyroid adenoma after coronary artery bypass surgery.* Ann Thorac Surg, 2005. **80**(3): p. 1105-6.
212. Morimoto, H., et al., *Decrement in bone mineral density after parathyroidectomy in a pediatric patient with primary hyperparathyroidism.* Clin Pediatr Endocrinol, 2018. **27**(2): p. 81-86.

213. Imachi, H., et al., *Ectopic mediastinal parathyroid adenoma: a cause of acute pancreatitis.* Endocrine, 2009. **36**(2): p. 194-7.
214. Cakmak, H., et al., *Giant mediastinal parathyroid adenoma.* Tuberk Toraks, 2011. **59**(3): p. 263-5.
215. Hussain, R.A.H., et al., *Ectopic Parathyroid Incidentaloma on Tc99m SestaMIBI Myocardial Perfusion Imaging.* Indian J Nucl Med, 2017. **32**(3): p. 235-236.
216. Inabnet, W.B. and C.A. Chu, *Transcervical endoscopic-assisted mediastinal parathyroidectomy with intraoperative parathyroid hormone monitoring.* Surg Endosc, 2003. **17**(10): p. 1678.
217. Dinga Madou, I., G.G. Callender, and A.W. Kim, *Something old, something new: Marrying 2 approaches to resect an ectopic parathyroid adenoma.* J Thorac Cardiovasc Surg, 2016. **151**(2): p. e33-4.
218. Elhelf, I.A.S., et al., *Ectopic mediastinal parathyroid adenoma localized with four-dimensional CT: a case report.* Radiol Case Rep, 2017. **12**(2): p. 247-250.
219. Bodner, J., et al., *First experiences with the da Vinci operating robot in thoracic surgery.* Eur J Cardiothorac Surg, 2004. **25**(5): p. 844-51.
220. Shah-Patel, L.R., et al., *Gamma Probe Detection of Ectopic Parathyroid Adenoma.* Radiol Case Rep, 2008. **3**(1): p. 161.
221. Hofbauer, L.C., et al., *Mediastinal Parathyroid Tumor: Giant Adenoma or Carcinoma?* Endocr Pathol, 1997. **8**(2): p. 161-166.
