# Supplementary material for: Mediastinal Parathyroid Cancer
Source: Cancers (Basel). 2022 Nov 28;14(23):5852. doi: 10.3390/cancers14235852 (PMC9739626; doi:10.3390/cancers14235852)
Supplement: Supplementary file 1 [file cancers-14-05852-s001.zip › cancers-2038037-MPC_Supplementary Table S1-revised.pdf]

Supplementary Table S1: Clinical and surgical outcomes of mediastinal parathyroid carcinoma

Author	Year of pub	Age at Diagnosis (years)	Gender	Ca (mM)	PTH (xUNL)[a]	size of lesion (mm)	Surgery	No. lymph nodes dissected	Lymph node status	Recurrence	time to recurrence (months)	Recurrence type	DOD	overall follow-up (months)	Vascular invasion	Resection margins
Weissman[1]	1956	46	M	3.8	nk	155	LE	nk		yes	48	Local+distant	yes	84	nk	
Lee [2]	1974	45	F	3.7	nk	60	LE	nk		nk			nk		no	
van Heerden[3]	1979	60	F	4.0	nk	nk	LE	nk		persistent	0	Local	yes	36	nk	
Chahinian [4]	1981	69	F	nk	nk	150	LN biopsy	nk	positive	persistent	0	Local	nk		nk	
Murphy [5]	1986	51	M	nk	nk	nk	LE[c]	nk		persistent	0	Local	nk		yes	
Kastan [6]	1987	57	F	3.7	3.8	53	LE	nk		no			no	84	yes	
Putnam [7]	1990	47	M	4.4	18.6	nk	LE	2	0/2	no			no	8	nk	
Kelly [8]	1994	62	F	3.6	4.9	30	LE	nk		no			no	24	yes	
Boddi [9]	1994	56	M	3.6	18.8	40	*en bloc*+LN	nk	positive	nk			nk		nk	
Favia (case ser)[10]	1998	72	M	4.2	nk	30	LE	nk		yes	9	Local+distant	yes	11	nk	
Favia (case ser)[10]	1998	56	M	4.1	nk	40	LE	nk		yes	29		yes	29	nk	nk
Delaney [11]	1999	44	M	4.1	3.3	50	LE	nk		no			no	12	yes	
Yamashita [12]	2001	43	F	3.5	16.9	nk	LE	nk		yes	84	Local	no	84	nk	
Dionisi [13]	2002	35	M	3.9	12.8	30	LE	nk		yes	24	Local	yes	24	nk	
Chiofala [14]	2005	66	M	3.6	28.1	120	*en bloc*+LN	nk	positive	no			no	12	yes	
Moran (case ser)[15]	2005	nk	nk	nk	nk	nk	nk	nk		nk			nk		nk	
Moran (case ser)[15]	2005	nk	nk	nk	nk	nk	nk	nk		nk			nk		nk	
Damadi [16]	2007	66	F	2.8	2.1	30	*en bloc*+LN	nk	negative	nk			nk		yes	
Vazquez [17]	2007	84	F	2.8	3.5	60	LE[c]	nk		persistent	0	Local	nk		nk	
Agha [18]	2007	65	F	3.9	7.0	45	LE	nk		nk			nk		nk	
Righi [19]	2008	10	M	3.8	4.3[b]	25	LE	nk		no			no	24	yes	
Iwata [20]	2008	61	M	1.9	18.8	85	LE	nk		no			no	36	nk	
Yong [21]	2010	23	M	3.6	47.8	36	LE	nk		no			no	24	nk	
Meng [22]	2011	28	M	5.8	36.0	37	en bloc	nk		no			no	12	yes	
Gawrychowski [23]	2012	72	F	nk	12.4	25	LE	nk		nk			nk		nk	
Ferri [24]	2012	76	F	3.3	16.9[b]	50	*en bloc*+LN	nk	negative	yes	24	Local	no	39	nk	
Tseng [25]	2013	72	M	3.5	3.4	20	*en bloc*	nk		no			no	36	yes	
Pesovic [26]	2013	54	M	3.4	18.1[b]	60	LE	nk		nk			nk		nk	
Cao [27]	2015	65	F	3.4	40.5	50	LE	nk		no			no	36	nk	
Pyzik [28]	2016	49	M	3.6	26.4	nk	LE	nk		persistent	0	Local	yes	10	nk	
Lu [29]	2017	65	M	3.6	27.4	35	LE	nk		no			no	1	nk	
Our case		45	M	2.9	13.5	37	*en bloc*+LN	28	0/28	no			no	37	yes	R0
Mean		54.8±16.4		3.6±0.7	16.8±12.8	54.8±37.0	*en bloc*: 7			yes: 6	22.3±15.2	local: 8	yes: 6	31.6±24.7	yes: 10	
Median		56.5	F: 12	3.6	16.9	42.5	LE: 20			no: 13	24	local+distant: 2	no: 15	24	no: 1	R0: 1
Range		10-84	M: 18	1.9-5.8	2.1-47.8	20-155	other: 3			persistent: 5	0-84	10		1-84		
Total with data available (n)	32	30	30	25	23	24	30	30	?/30	24	10		21	21	11	1

nk; data not known, [a]; times upper normal limit, [b]; calculation based on assumed upper normal limit of 70ng/l , [c]; partial excision as tumour was difficult to excise, LN: lymph node dissection, DOD; Dead of disease

1. Weissman, I., J.P. Worden, and J.M. Christie, *Mediastinal parathyroid carcinoma with metastases; report of a case and review of the literature.* Radiology, 1957. **68**(3): p. 352-7.
2. Lee, Y.T. and J.K. Hutcheson, *Mediastinal parathyroid carcinoma detected on routine chest films.* Chest, 1974. **65**(3): p. 354-5.
3. van Heerden, J.A., et al., *Cancer of the parathyroid glands.* Arch Surg, 1979. **114**(4): p. 475-80.
4. Chahinian, A.P., et al., *Metastatic nonfunctioning parathyroid carcinoma: ultrastructural evidence of secretory granules and response to chemotherapy.* Am J Med Sci, 1981. **282**(2): p. 80-4.
5. Murphy, M.N., et al., *Nonsecretory parathyroid carcinoma of the mediastinum. Light microscopic, immunocytochemical, and ultrastructural features of a case, and review of the literature.* Cancer, 1986. **58**(11): p. 2468-76.
6. Kastan, D.J., et al., *Carcinoma in a mediastinal fifth parathyroid gland.* Jama, 1987. **257**(9): p. 1218-9.
7. Putnam, J.B., Jr., et al., *Extended en bloc resection of a primary mediastinal parathyroid carcinoma.* Ann Thorac Surg, 1990. **50**(1): p. 138-40.
8. Kelly, M.D., et al., *Parathyroid carcinoma in a mediastinal sixth parathyroid gland.* Aust N Z J Surg, 1994. **64**(6): p. 446-9.
9. Boddi, W., et al., *[Hyperparathyroidism due to parathyroid carcinoma located in the mediastinum].* Ann Ital Med Int, 1994. **9**(1): p. 32-4.
10. Favia, G., et al., *Parathyroid carcinoma: sixteen new cases and suggestions for correct management.* World J Surg, 1998. **22**(12): p. 1225-30.
11. Delaney, S.E., et al., *Mediastinal parathyroid carcinoma.* Endocr Pract, 1999. **5**(3): p. 133-6.
12. Yamashita, K., et al., *A case of familial isolated hyperparathyroidism with ectopic parathyroid cancer.* Endocr J, 2001. **48**(4): p. 453-8.
13. Dionisi, S., et al., *Concurrent parathyroid adenomas and carcinoma in the setting of multiple endocrine neoplasia type 1: presentation as hypercalcemic crisis.* Mayo Clin Proc, 2002. **77**(8): p. 866-9.
14. Chiofalo, M.G., et al., *Huge parathyroid carcinoma: clinical considerations and literature review.* World J Surg Oncol, 2005. **3**: p. 39.
15. Moran, C.A. and S. Suster, *Primary parathyroid tumors of the mediastinum: A clinicopathologic and immunohistochemical study of 17 cases.* American Journal of Clinical Pathology, 2005. **124**(5): p. 749-754.
16. Damadi, A., et al., *Use of pre-operative Tc99m-Sestamibi scintigraphy and intraoperative parathyroid hormone monitoring to eliminate neck exploration in mediastinal parathyroid adenocarcinoma.* J Surg Educ, 2007. **64**(2): p. 108-12.
17. Vazquez, F.J., et al., *Parathyroid carcinoma presenting as a giant mediastinal retrotracheal functioning cyst.* Singapore Med J, 2007. **48**(11): p. e304-7.
18. Agha, A., et al., *Parathyroid carcinoma in multiple endocrine neoplasia type 1 (MEN1) syndrome: two case reports of an unrecognised entity.* J Endocrinol Invest, 2007. **30**(2): p. 145-9.
19. Righi, A., K. Dimosthenous, and J. Mize, *Mediastinal parathyroid carcinoma with tumor implants in a child: a unique occurrence.* Int J Surg Pathol, 2008. **16**(4): p. 458-60.
20. *First treatment for serious complications of kidney disease, parathyroid cancer.* FDA consumer, 2004. **38**(3): p. 6.
21. Yong, T.Y. and J.Y. Li, *Mediastinal parathyroid carcinoma presenting with severe skeletal manifestations.* J Bone Miner Metab, 2010. **28**(5): p. 591-4.
22. Meng, Z., et al., *Ectopic parathyroid carcinoma presenting with hypercalcemic crisis, ectopic uptake in bone scan and obstruction of superior vena cava.* Clin Nucl Med, 2011. **36**(6): p. 487-90.
23. Gawrychowski, J., et al., *Mediastinal parathyroid carcinoma: a case report.* Endokrynol Pol, 2012. **63**(2): p. 143-6.
24. Ferri, E., et al., *Hyperfunctional parathyroid carcinoma with mediastinal extension.* Acta Otorrinolaringol Esp, 2012. **63**(1): p. 68-71.
25. Tseng, C.W., et al., *Ectopic mediastinal parathyroid carcinoma presenting as acute pancreatitis.* J Chin Med Assoc, 2013. **76**(2): p. 108-11.
26. Pesovic, J.P., et al., *Cancer of ectopic parathyroid gland presentation of the disease with a case report.* Int J Clin Exp Med, 2013. **6**(3): p. 227-30.
27. Cao, J., et al., *Parathyroid carcinoma: A report of six cases with a brief review of the literature.* Oncol Lett, 2015. **10**(6): p. 3363-3368.
28. Pyzik, A.J., et al., *Parathyroid cancer - difficult diagnosis - a case report.* Nucl Med Rev Cent East Eur, 2016. **19**(1): p. 46-50.
29. Lu, C., et al., *Superior mediastinal typical carcinoid detected by 99mTc-MIBI SPECT/CT imaging: A case report.* Medicine (Baltimore), 2017. **96**(52): p. e9457.
