# Supplementary material for: Mediastinal Parathyroid Cancer
Source: Cancers (Basel). 2022 Nov 28;14(23):5852. doi: 10.3390/cancers14235852 (PMC9739626; doi:10.3390/cancers14235852)
Supplement: Supplementary file 1 [file cancers-14-05852-s001.zip › cancers-2038037-MPC_Supplementary Table S2-revised.pdf]

| Supplementary Table S2<br>The inverse 3+3 criterion, small lesion with moderate hypercalcemia, predicts **absence** of MPC<br>P= has no cancer = 257; N= has cancer = 23 | | | | | | |
|---|---|---|---|---|---|---|
| | Σ test pos | sensitivity TP/P | specificity TN/N | PPV TP/(TP+FP) | NPV TN/(TN+FN) | accuracy (TP+TN)/(P+N) |
| n=280 | | | | | | |
| <3.0 cm AND <3.0 mM | 129 | 129/257<br>50.2% | **23/23**<br>**100%** | **129/129**<br>**100%** | 23/151<br>15.2% | 151/280<br>30.5% |
